# Supplementary material for: A web-database of mammalian morphology and a reanalysis of placental phylogeny
Source: BMC Evol Biol. 2007 Jul 3;7:108. doi: 10.1186/1471-2148-7-108 (PMC1941728; doi:10.1186/1471-2148-7-108)
Supplement: Additional File 1 — Website data: The 196 characters first described in [17], along with the supplementary data indicated below, have been joined into a number of HTML files, all of which are linked to the "morphsite_bmc07.html" index page. The embedded links to the supporting files are functional, independent of PC or platform, provided that the directory structure remains intact (i.e., all files remain in a single folder, no subdirectories). Nexus files: Datafiles containing the morphological, indel, and aligned DNA sequence data, linked from the "morphsite_bmc07.html" index page. Table 1. Changes to the multiple sequence alignment of [9]. Table 2. Qualitative character-state corrections to the morphology matrix of [17]. Table 3. Summary of discrepancies between Appendices 1 and 2 of [17]. [file 1471-2148-7-108-S1.zip › asher-BMC07-website/teeth.html]

asher-characterBank


|  |  |  |
| --- | --- | --- |
| Dentition and  Mandible |  | This work is licensed under a   Creative Commons License. |
| Upper Dentition  94 dental reduction  ;  0 teeth present 1 atrophied 2 baleen,   95 incisor enamel  ;  0 covers tooth 1 anterior band 2 abs-tip only,   96 number of incisors;  0 none 1 one 2 two 3 three 4 four 5 five,   97 posterior premaxillary diastema  ;  0 small-abs 1 large gap,   98 incisor growth ;  0 crown-root junction 1 hypsodont,   99 tooth replacement ;  0 antemolars 1 last premolar only 2 uncalcified 3 continuous,   100 canine growth ;  0 determinate 1 hypsodont,   101 cheektooth enamel ;  0 present 1 atrophied,   102 cheektooth orientation  ;  0 parallel 1 V-shaped,   103 cheektooth growth  ;  0 crown-root suture 1 hypsodont,   104 upper P3  ;  0 pres 1 abs,   105 P3 crown ;  0 mediolaterally compressed 1 prominent lingual cusp(s),   106 P4 crown ;  0 mediolaterally compressed 1 prominent lingual cusp(s),   107 metacone ;  0 present 1 absent,   108 protocone ;  0 present 1 absent,   109 molar ectoloph  ;  0 unconnected 1 pi shaped,   110 stylar shelf ;  0 broad 1 narrow,   111 molar shearing ;  0 multilocus 1 carnassial, | Jaw  112 mandible ;  0 robust 1 threadlike,   113 condyle height  ;  0 at toothrow 1 above toothrow,   114 coronoid shape  ;  0 large 1 reduced,   115 coronoid position  ;  0 superior condyle 1 inferior condyle,   116 jaw angle  ;  0 narrow 1 medial inflection,   117 symphysis-fusion  ;  0 unfused 1 fused,   118 symphysis-length  ;  0 anteroposteriorly narrow 1 >30% dentary length,   119 symphysis-ventral lip  ;  0 absent 1 anteroventral lip, | Lower Dentition 120 lower m3 presence ;  0 present 1 absent,   121 talonid basin  ;  0 present 1 reduced, |
|  |  |  |
